# Pollution Vulnerability of the Ghiss Nekkor Alluvial Aquifer in Al-Hoceima (Morocco), Using GIS-Based DRASTIC Model

**DOI:** 10.3390/ijerph20064992

**Published:** 2023-03-12

**Authors:** Yassine El Yousfi, Mahjoub Himi, Mourad Aqnouy, Said Benyoussef, Hicham Gueddari, Imane Lamine, Hossain El Ouarghi, Amar Alali, Hanane Ait Hmeid, Mohamed Chahban, Abdennabi Alitane, Abdallah Elaaraj, Kamal Abdelrahman, Tamer Abu-Alam, Ali Ait Boughrous, Azzeddine Khafouri, Mohamed Abioui

**Affiliations:** 1 Water and Environment Management, Laboratory of Applied Sciences (LSA), National School of Applied Sciences Al Hoceima, Abdelmalek Essaadi University, Tétouan 93030, Morocco; 2 Mineralogy, Petrology and Applied Geology, University of Barcelona, 08028 Barcelona, Spain; 3 AGRL, AGRSRT, Department of Geosciences, Faculty of Sciences and Techniques, Moulay Ismail University of Meknes, Errachidia 52000, Morocco; 4 Research Team of Biology, Environment and Health, Department of Biology, Faculty of Science and Technology, University of Moulay Ismail, Errachidia 52000, Morocco; 5 OLMAN BPGE Laboratory, Multidisciplinary Faculty of Nador, Mohamed First University, Oujda 60000, Morocco; 6 Laboratory of Aquatic Systems, Marine and Continental Environments (AQUAMAR), Department of Biology, Faculty of Sciences, Ibnou Zohr University, Agadir 80000, Morocco; 7 Geoengineering and Environment Laboratory, Research Group “Water Sciences and Environment Engineering”, Geology Department, Faculty of Sciences, Moulay Ismail University, Meknes 50000, Morocco; 8 Hydrology and Hydraulic Engineering Department, Vrije Universiteit Brussels (VUB), 1050 Brussels, Belgium; 9 Natural Resources and Environment Laboratory, Geology Department, Polydisciplinary Faculty of Taza, Sidi Mohamed Ben Abdellah University, Taza 35000, Morocco; 10 Engineering Sciences and Techniques Center, Environment Department, Faculty of Science and Technology, Sidi Mohamed Ben Abdellah University, Fez 30000, Morocco; 11 Department of Geology & Geophysics, College of Science, King Saud University, Riyadh 11451, Saudi Arabia; 12 The Faculty of Biosciences, Fisheries and Economics, UiT the Arctic University of Norway, 9037 Tromsø, Norway; 13 Laboratory of Geoheritage, Geoenvironment and Prospecting of Mines &Water, Department of Earth Sciences, Faculty of Sciences, Mohammed Premier University, Oujda 60000, Morocco; 14 Department of Earth Sciences, Faculty of Sciences, Ibnou Zohr University, Agadir 80000, Morocco; 15 MARE-Marine and Environmental Sciences Centre—Sedimentary Geology Group, Department of Earth Sciences, Faculty of Sciences and Technology, University of Coimbra, 3030-790 Coimbra, Portugal

**Keywords:** vulnerability, DRASTIC model, groundwater pollution, nitrate, Ghiss Nekkor alluvial aquifer

## Abstract

Groundwater resources of the alluvial aquifer Ghiss Nekkor, which covers an area of 100 km^2^, are the main source of domestic and agricultural freshwater supply in the region of Al Hoceima in Morocco. Due to human activities (overexploitation, increase in agricultural activity), this alluvial aquifer has become very sensitive to chemical pollution. The principal objective of this current study is to develop and implement a calibration method to assess, map, and estimate the vulnerability of the Ghiss Nekkor alluvial aquifer to pollution risk. In this work, the GIS-based DRASTIC model was used to estimate the inherent vulnerability to contamination of the Ghiss Nekkor alluvial aquifer with seven standard hydrogeological parameters. Nitrate (NO_3_) and electrical conductivity (EC) data were used to validate the DRASTIC map. The results of the vulnerability map analysis show that the vulnerability to contaminants varies from non-existent in the southwestern part of the plain (7.3% of the total area), to very high (14.5%). The vulnerability is moderate in the central and northeastern areas (26.9%), while it is high in the other areas (17.5%). Furthermore, the most sensitive areas are mainly concentrated near the coastal strip and the central plain on both sides of the Nekkor River. In these areas, the NO_3_ and EC values are above the maximum allowable limit of the World Health Organization. The results suggest that the DRASTIC model can be an effective tool for decision-makers concerned about managing groundwater sustainability.

## 1. Introduction

Water demand has been increasing globally, and satisfying the demand is becoming a serious challenge [1,2,3,4,5]. In Morocco, economic and social development depends on groundwater resources. Most of the urban and rural areas of the country rely on water sources for drinking, irrigation, and industry [6]. Within Al Hoceima province, the Ghiss Nekkor alluvial aquifer is a significant source of water. This coastal aquifer has been under high stress from excessive groundwater abstraction, wastewater discharge, seawater intrusion, and point-source contamination [7,8,9]. The deterioration of groundwater quality is a significant factor in the water scarcity problem, particularly when it is used for drinking water. Therefore, groundwater must be protected against the growing threat of contamination [10]. The quality is generally subject to considerable potential for pollution, especially in areas dominated by agriculture, with strenuous activities implying the utilization of pesticides and fertilizers [11,12,13,14,15,16]. Additionally, it is necessary to analyze the quality of wastewater before releasing it and implement a cost-effective method for avoiding and controlling groundwater pollution [17,18].

As compared to surface water, groundwater is currently the purest drinking water source [19]. Groundwater’s chemical quality has been altered due to the extensive usage of aquifers. The investigation of these changes requires continuous monitoring. Vulnerability maps have also become an increasingly necessary tool for environmental management and groundwater protection [20]. The use of these maps is considered among the most effective tools for the monitoring system. Consequently, vulnerability assessment might be utilized for various activities, including groundwater resource management, decision-making, land use planning, and groundwater quality maintenance [21]. Alternatively, vulnerability models, including the mechanisms of transport, retention, and attenuation of pollutants in the aquifer, may be used to evaluate vulnerability [22].

The DRASTIC approach is considered one of the major models used in groundwater vulnerability evaluation [23]. Nevertheless, to produce a reasonably accurate result, the DRASTIC approach must be adapted to meet the specific hydrogeological demands of the region in which it is used [24]. To examine and evaluate the vulnerability of groundwater in the Ghiss Nekkor alluvial aquifer to pollution risk, many methodologies have been developed [23,25,26,27,28,29,30,31,32,33]. However, there is no standard methodology for evaluating and validating an aquifer approach. Several researchers have attempted to correlate the vulnerability index with data describing chemicals or contaminants [34,35,36]. Additional researchers have related land use to vulnerability [37,38], without access to the rate correction of the DRASTIC model.

The novelty of this study is the exploitation of recent input data (piezometric level, precipitation, lithological section of the boreholes), as well as the appropriate results of recent physicochemical analyses (NO_3_ and EC) carried out in the laboratory. Since nitrate does not generally exist under natural conditions in the groundwater, especially in agricultural areas, it can be a valuable indication of pollution spreading from the surface to the groundwater level [39]. This research aims to apply an existing vulnerability estimation method to the Ghiss Nekkor alluvial aquifer (100 km^2^) using the DRASTIC model with NO_3_ and EC measurements. The specific goals of this study are to calibrate and implement the method to assess, map, and estimate the groundwater vulnerability in the aquifer.

## 2. Study Area

**Figure 1:**
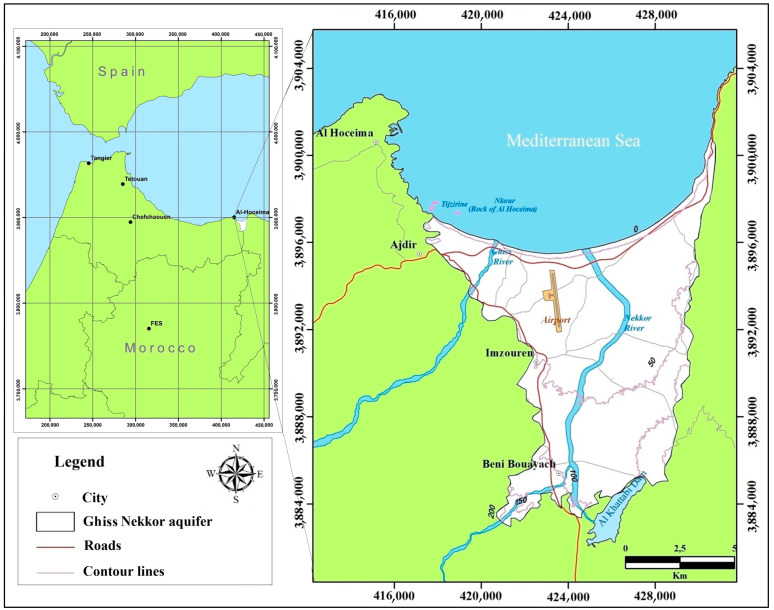
Geographical location of the Ghiss Nekkor aquifer.

The Ghiss Nekkor plain is located in the northeast of Morocco and contains a multi-layered alluvial aquifer (100 km^2^); it is considered the most important alluvial aquifer in the Moroccan Mediterranean region [40]. The aquifer is situated between longitudes of 35°3.53′ and 35°15.05′ west and latitudes of 3°55.11′ and 3°45.42′ north (Figure 1). The Plio-Quaternary formation, forming the main part of the Ghiss Nekkor alluvial plain, is represented by detrital materials with lateral and vertical variations in lithological facies. The water table is free or captive, depending on the sector. The maximum elevation in the study area is 231 m above sea level, while the lowest point is 5 m above sea level. The recharge comes from the underflow of the Nekkor and Ghiss rivers, and infiltration of surface water; pumping and discharge to the ocean are the main outlets. The transmissivity of the water table varies between 0.1 and 6.4 ×10^−2^ m^2^/s [40].

The climate is typically Mediterranean with a trend towards semiarid. It is usually hot and dry in summer and cold and wet in winter. The climate of the Nekkor watershed is characterized by irregular rainfall, as well as a significant evapotranspiration potential. The average annual precipitation ranges from 286.4 mm to 311.5 mm among the different stations contained in the basin, but the average interannual rainfall of the basin is estimated to be 340 mm [41]. The temperature averages show generally strong variations owing to remarkable seasonal variations, with an extremely cold winter and an extremely hot summer. The average annual temperature exceeds 30 °C [42,43].

The groundwater has a total salinity of 1.5 to 3.5 g/1 with a predominance of sodium chloride–sulfate facies. The water table in the plain’s lowest reaches is less than 5 m deep. This depth increases rapidly towards the SE, and E. Being able to vastly exceed 50 m at the very S of the plain, the depths decrease again, correlating with a rise in the impermeable substratum. A total of 55 km^2^ is covered by areas where the water table is even less than 20 m deep [40].

Artificial withdrawals are almost nil (borehole 251/5: 20 L/s for the water supply of Al-Hoceima); the only hidden withdrawals are due to evaporation in the downstream part of the water table, close to the ground (about 130 L/s). Total withdrawals are, therefore, about 150 L/s. The terms of the balance of this water table have shown that the withdrawals made are mainly intended for irrigation and drinking water supply to populations in rural areas. Additionally, part of the aquifer’s water is lost by evaporation in the downstream part where the water levels are less than 5 m from the ground level. The rest of the resources are lost to the sea.

**Figure 2:**
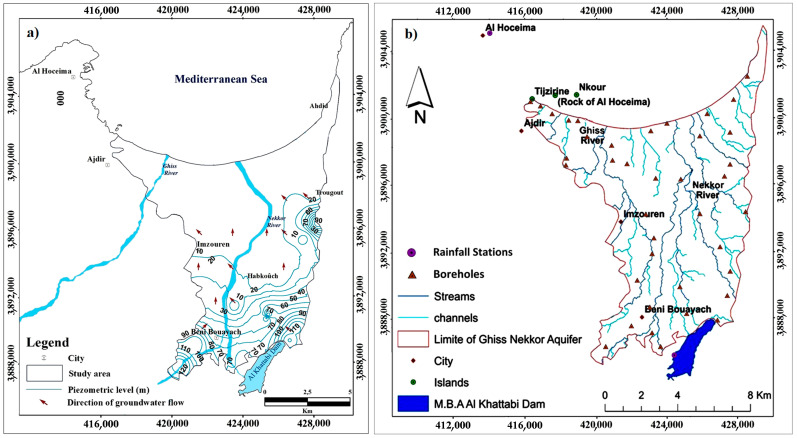
(**a**) Piezometric map and (**b**) hydrographic network of the Ghiss Nekkor aquifer.

The project site belongs to the Rif chain, where the principal geological formations encountered surrounding the Ghiss Nekkor alluvial aquifer are the limestones (Lias-Cretaceous) of the internal dorsal, the marls and sandstones of the Ajdir aquifer, and alluvium and silts of the Middle and Recent Quaternary [44,45]. The studied region includes two main units: Ghiss and Nekkor; the Ghiss Nekkor watershed comprises a moderately dense hydrographic network oriented from the south to the north that shelters the Mediterranean Sea. The main rivers that drain the study area are the River Ghiss and the River Nekkor. The global geometric configuration of the Ghiss Nekkor watershed is presented in Figure 2. 

## 3. Materials and Methods

**Figure 3:**
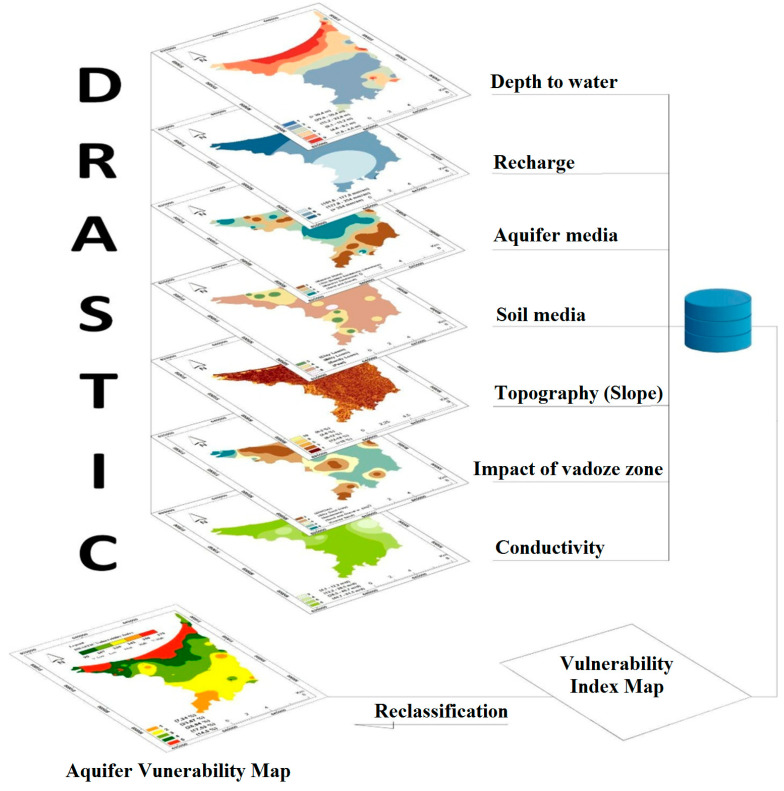
Methodology flowchart for the vulnerability analysis of the groundwater of the Ghiss Nekkor aquifer using the DRASTIC approach in a GIS.

The GIS-based DRASTIC method was employed to analyze groundwater intrinsic vulnerability based on seven hydrogeological characteristics that might impact pollution transport via vadose zone to aquifers [23,24]. These parameters are depth to water (parameter D), recharge or infiltration (parameter R), type of aquifer (parameter A), type of soil (parameter S), topography (parameter T), nature of the vadose zone (parameter I), and the hydraulic conductivity (parameter C). Each parameter is attributed a score (r_i_) and unique weight (w_i_). According to their relative significance in evaluating contamination potential, weight factors vary from 1 to 5. Data rating can vary by type, range, and frequency, allowing the model to operate in a site-specific manner. The flowchart of the methodology and approaches used is presented in Figure 3. The linear additional combination of the previously mentioned parameters, including weights and ratings, was applied to generate the (DVI) DRASTIC vulnerability index shown below [23]:

$$\mathrm{DVI}=D_{r}\cdot D_{w}+R_{r}\cdot R_{w}+A_{r}\cdot A_{w}+S_{r}\cdot S_{w}+T_{r}\cdot T_{w}+I_{r}\cdot I_{w}+C_{r}\cdot C_{w}$$

The depth to water (parameter D) in the Ghiss Nekkor alluvial aquifer was determined from piezometers located in this aquifer, and by using the IDW interpolation technique on 73 wells; aquifer depth data have been spatially transformed. Net recharge (parameter R) or annual net recharge measures the quantity of water that enters the saturated zone during a specific period, i.e., the transfer of contaminants to the groundwater. Net recharge means the fringe of water that infiltrates the earth and accesses the aquifer. It facilitates the vertical transport of pollutants to the aquifer [46]. The data used in our case are the average annual rainfall data interpolated from seven meteorological stations in the region. We used the IDW method on the seven stations under ArcGis 10.5 to transform these curves into a net recharge. The type of aquifer (parameter A) is derived from geological and hydrogeological surveys of the Ghiss Nekkor plain. Thus, the vulnerability index will be higher if the aquifer is made of highly porous materials. The type of soil (parameter S) was derived from Rif’s pedological map and refers to the upper section of the vadose zone. Topography (parameter T) was generated from a 30 × 30 m digital elevation model. The impact of the vadose zone and hydraulic topography (parameter I) was generated from the borehole lithological section data included in the study area. Hydraulic conductivity (parameter *C*) was determined using pumping tests in the Ghiss Nekkor plain.

The unique assessment classes altered the DRASTIC approach for each parameter relevant to the research area [23]. The five-class land use map was selected to reflect the many methods that may contribute to groundwater pollution.

In addition, nitrate concentration was the principal pollution parameter selected to calibrate the DRASTIC model. Samples are collected from seventy-three wells and analyzed. The precise location of each well was obtained through GPS techniques. Nitrate percolates from the surface layer in most cases under normal conditions. Thus, it may be used as an indication to show if the vulnerability index accurately reflects actual needs in the region [47]. The following essential conditions must be satisfied to apply nitrate to optimize weights:

- The combination of comparatively shallow groundwater depths.
- The principal source of nitrate must be derived from agricultural operations conducted on the surface.
- Nitrate leaching must be caused by surface water recharging for long periods to correlate pollution with human activities.
- The surface distribution must be generally uniform.

During our field sampling in May 2018, we used a multiparameter device (HANA HI 98194) to measure temperature (T), pH, total dissolved solids (TDS), and electrical conductivity (EC) in situ immediately after sample collection. The device has an accuracy of ±0.01 units for pH and ±1% (or ±1 μS/cm) for electrical conductivity. To ensure accurate chemical analysis, we filtered the samples in the field using a 0.45 μm filter before dividing them into two bottles—one for anions and the other for acidified cations. For wells equipped with a pump, samples were taken after pumping to ensure representative sampling.

Nitrate concentrations collected and surveyed in May 2018 were utilized to calibrate the index, as well as additional measurements from August 2018, which were used to determine the correlation factor. The laboratory analysis of nitrate was conducted using a Shimadzu UV-1800 spectrophotometer at the LSA-GE2 laboratory of ENSA Al Hoceima. To compute the change rate of each DRASTIC parameter, the nitrate measurements were classified into six classes, and the average of each class was used. It should be noted that the same chemical data used in this study were previously published by El Yousfi et al. [40].

The resulting value is a relative measurement of vulnerability to pollution; areas with a high rate index are considered more vulnerable than those with a significantly lower value. The parameter rates and weights used in the original DRASTIC approach are described in Persson et al. [23].

Data sources used for preparing the vulnerability map of the Ghiss Nekkor alluvial aquifer were:

- Piezometric level of wells collected during a field mission in May 2018.
- Geological and pedological data of the Rif.
- Storage coefficient, precipitation, recharge, and hydraulic conductivity “The Loukkos Water Basin Agency (ABHL)”.
- Nitrate concentrations based on the analysis of water samples collected in May 2018.
- Lithology data of unsaturated and saturated zones covering the study area extracted from boreholes archived by the Loukkos Water Basin Agency (ABHL).

## 4. Results and Discussion

### 4.1. Model Results

#### 4.1.1. Depth to Water (D)

**Figure 4:**
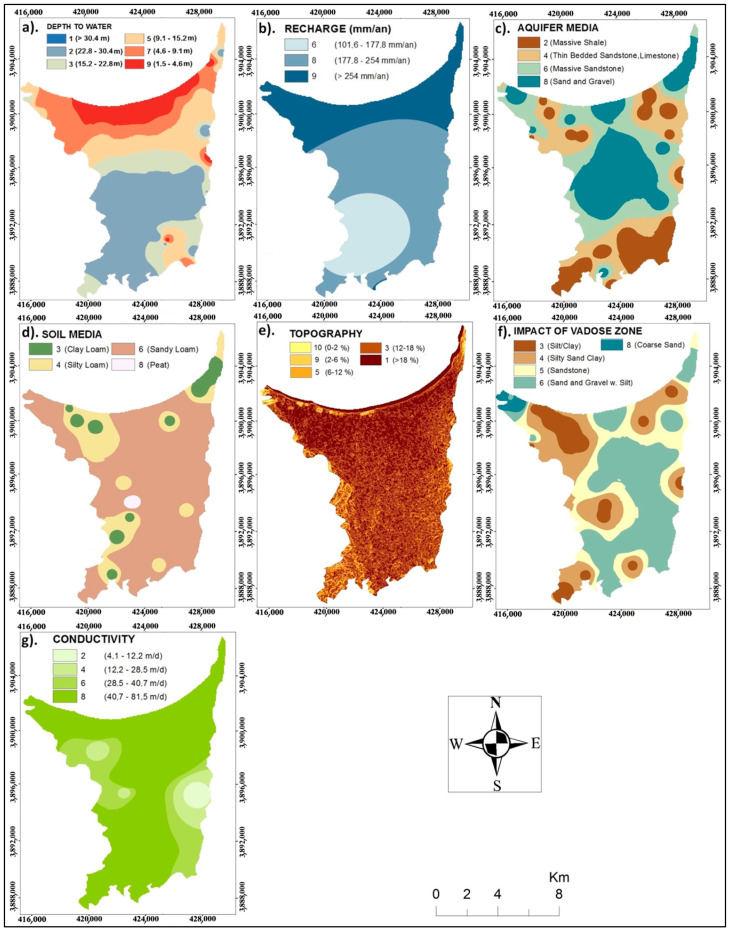
Maps showing the integration of (**a**) depth to water, (**b**) recharge, (**c**) aquifer media, (**d**) soil media, (**e**) topography (slope), (**f**) impact of vadose zone, and (**g**) conductivity.

In general, the protective capacity of an aquifer is directly proportional to its depth, which means that a deeper aquifer has a greater potential to provide protection [48]. The spatial variability of the depth to the aquifer is represented by six classes ranging from 1.57 to 69.5 m (Figure 4a). The majority of the aquifer is characterized by class 1 and is located above 34 m in depth. The depth to the water map of the Ghiss Nekkor alluvial aquifer presents one of the most significant parameters to assess pollution vulnerability. It also provides an overview of the distance the contaminant must travel before arriving at the aquifer. Thus, the risk of contamination is essential when the water level in the aquifers increases.

#### 4.1.2. Net Recharge (R)

The study area is bordered by numerous river systems and wetlands, and is primarily agricultural land. This area is recharged by rivers, rainfall, wetlands, and irrigation water. The higher the recharge rate, the greater the risk of contamination. To calculate the net recharge, we used the method of equations [49] related to the nature of the geological formations. Based on the widely used classification for hydrological soil group developed by the USDA [50,51], our analysis reveals that the study area is predominantly divided into two hydrological groups, B and C (Table 1).

The spatial variations of recharge are exposed in three classes. According to the DRASTIC model, the recharge parameter ratings vary between 6 and 9 (Figure 4b). The net recharge obtained over the entire study area is between 130 and 410 mm/year.

#### 4.1.3. Type of Aquifer (A)

This factor determines the aquifer’s groundwater discharge path. Attenuation capacity decreases when permeability increases, and vice versa [52]. The development of the map of the aquifer type was based, essentially, on the interpretation and correlation of more than 90 existing boreholes in the zone area (Figure 4c). These correlations show that the aquifer is formed essentially by sand and gravel, massive sandstone, and massive shale facies (Table 1).

#### 4.1.4. Type of Soil (S)

Soil characteristics control the downward movement of pollutants. Indeed, the existence of fine materials (silts and clays) and the organic matter value of the soil reduce the intrinsic permeability. The migration of the pollutants is delayed by the physicochemical processes of adsorption, oxidation, ionic exchange, and biodegradation [53,54]. The more clay-rich the soil, the more necessary it will be to preserve the groundwater, and the greater the protection of groundwater. Figure 4d illustrates the spatial distribution of soils in the study area. The soil types are weighted from 3 to 8.

#### 4.1.5. Topography (T)

For the topography parameter, the digital elevation model is a raster map collected from SRTM used for flow accumulation and slope percentage [55], which are assigned to the pixels based on the rating system of the DRASTIC method. This component influences the flow rate above the surface [55].

Therefore, the migration and infiltration of pollutants also increase in areas with a low slope. The raster map thus elaborated (Figure 4e) shows the predominance of the slope values ranging from 10 to 1 for the parameter rating.

#### 4.1.6. Impact of the Vadose Zone and Hydraulic Topography (I)

Borehole lithological sections are interpreted to determine the effect of the vadose area parameter during the assessment procedure. The correlation shows that clay facies, sand, and gravel constitute the unsaturated area with clay passages. The raster map elaborated according to the DRASTIC rating system shows the distribution of the vadose zone. It is obtained from digitizing geological maps with a 1/50,000 scale [56]. This type of data allows us to have a synthesis map showing the lithology of the area. We categorized the lithologic layers according to their infiltration degrees. The obtained classes are weighted from 3 to 8 (Figure 4f).

#### 4.1.7. Hydraulic Conductivity (C)

The hydraulic conductivity of the soil determines the rate at which water can move through the soil and into the aquifer, affecting the quantity of water that can percolate into groundwater. An aquifer with high conductivity is more sensitive to pollution since the amount of time it takes for the contaminated plume to move across it is increased [57]. The hydraulic conductivity values presented in this research are based on pumping test results. Hydraulic conductivity varied from 7.30 × 10^−5^ to 1.71 × 10^−3^ m/s in alluvium [58]. Table 2 and Figure 4g show the range and distribution of hydraulic conductivity values.

The ratings and weights associated with each DRASTIC characteristic are provided in Table 2, ranging from 1 to 10, with higher levels reflecting increased pollution levels.

### 4.2. Spatial Distribution of Vulnerability

The vulnerability analysis of the aquifer was accomplished as discussed in the methodology section. Using a combination of various parameters of the hydrogeological environment, a range of numerical values is generated, known as the DRASTIC index. The research area was represented by a composite layer that combines the component files of the grid given in Table 2.

**Figure 5:**
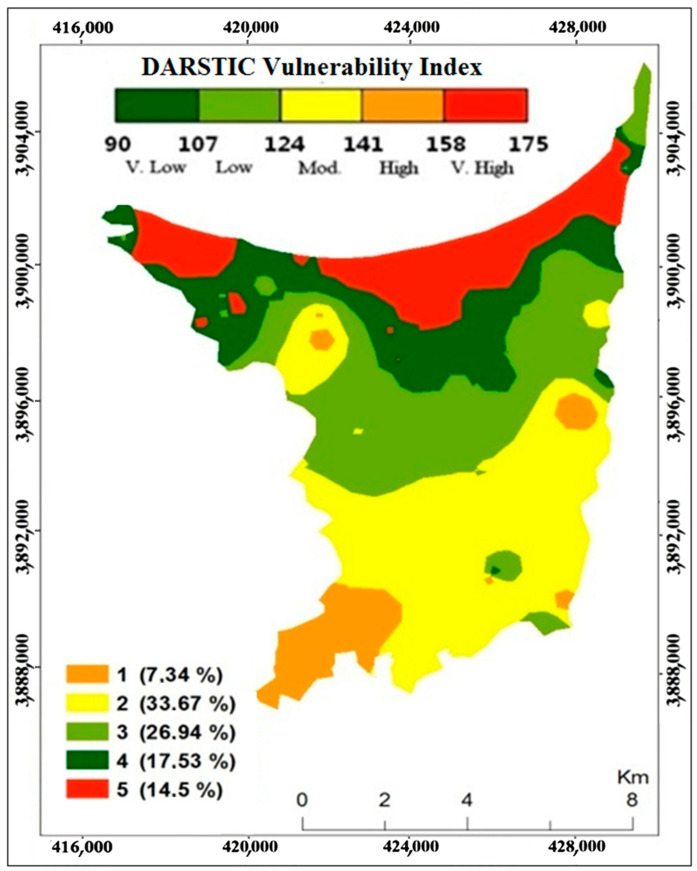
DRASTIC vulnerability index map.

According to the DRASTIC model index, the aquifer vulnerability is between 90 and 175, with an average of 137.7. The values were classified into five categories. They are very low (90–107), low (107–124), moderate (124–141), high (141–158), and very high (158–175). Groundwater vulnerability reveals the total percentage of surface area by each of the classes. In the northern part of the alluvial aquifer of Ghiss Nekkor (Figure 5), contamination susceptibility varies from high (17.5%) to very high (14.50% of the total area). These classifications are related to the sand and silt zone with a high potential for recharge, shallow water table, and permeable soils. These zones require particular consideration in determining future land use decisions. In the central and northeastern parts, the vulnerability to pollution is moderate (26.94%). The risk of pollution is very low in the southwest part (7.34% of the total area).

### 4.3. Validation of the Model Using Groundwater Chemistry

**Figure 6:**
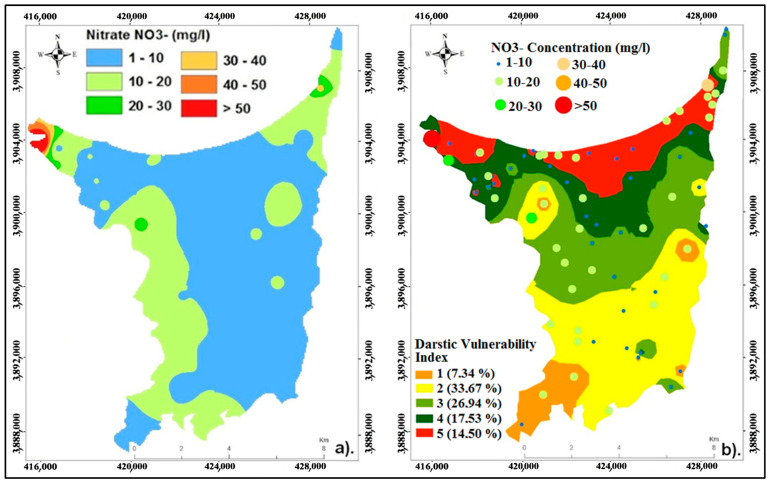
(**a**) Nitrate concentration distributions in the groundwater of Ghiss Nekkor, (**b**) vulnerability map and concentrations of nitrates for the study zone.

The spatial distribution concentrations of nitrate in the sample wells are shown in Figure 6a. The vulnerability map of the Ghiss Nekkor alluvial aquifer’s groundwater was validated using the groundwater’s nitrate content. The vulnerability ranking results are also synthesized along with the nitrate concentrations for each class (Figure 6b). Recent monitoring of 73 wells in the survey zone shows that nitrate content in about 8.22% of the total samples exceeded the limit authorized by the World Health Organization standard (50 mg/L). When the concentration of nitrates in the water exceeds 50 mg/L, it is classified as non-potable, since it is toxic for humans, particularly for the more vulnerable populations, such as pregnant women and children [55]. Comparing the DRASTIC map to the nitrate distribution map reveals a modest correlation between areas with increased nitrate concentrations and those with high vulnerability.

Additionally, the aquifer in the northwestern area is at risk of significant amounts of pollution caused by agricultural activities in sandy soil areas, where vast quantities of chemical fertilizers are used, in addition to the widespread usage of septic tanks in urban areas. High concentrations are further associated with culture zones where nitrate is applied as a chemical fertilizer. As a result, pollution can spread from manure [57,58]. Moving towards the sea, we observe the 0 m isopiestic line present inland, indicating the influence of marine intrusion in the salinization of the groundwater aquifer, as reported by El Yousfi et al. [40]. This can explain the high EC and TDS values observed along the coastline.

The present study, like those of other researchers [22,46,59], shows that DRASTIC-based vulnerability assessments can already be made coherent by allowing for the calibration of DRASTIC-based rate and weighting variables by a devoted evaluation of surveillance data sets. Similar to previous studies conducted by other researchers e.g., [22,46,59], the current study demonstrates that vulnerability assessments based on DRASTIC can be made more accurate by calibrating the rate and weighting variables of the DRASTIC method using dedicated evaluation of surveillance datasets. The calibrated DRASTIC parameters are predicted to be very specific for the primary research site. They are influenced by various monitoring data selections, land use, hydrogeological characteristics, and spatial resolution as reference contamination data.

**Figure 7:**
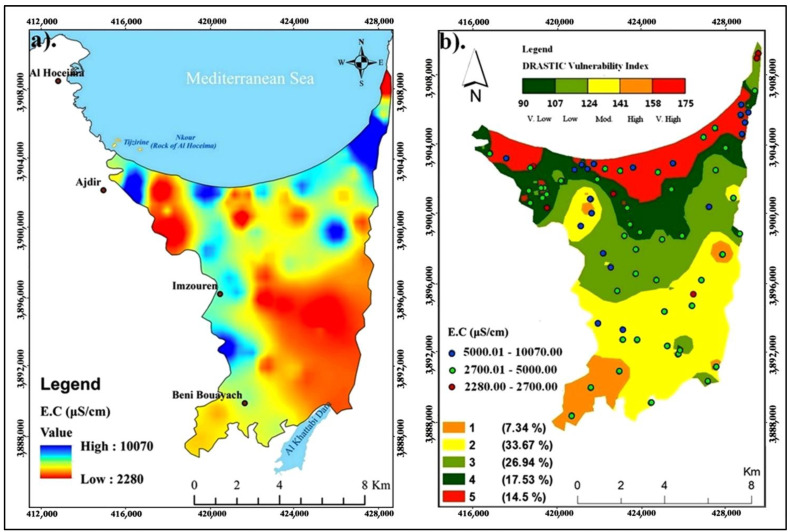
(**a**) Spatial distributions of EC in groundwater. (**b**) Original vulnerability map and EC conductivity for the study area.

The EC of groundwater samples of the Ghiss Nekkor alluvial aquifer varies from 2280 to 10,070 μS/cm. Furthermore, the high EC values are observed mainly in the northeast and northwest sectors of the aquifer. Thus, the high salt contents are marked in the southwest (Al Khattabi Dam) [58]. Increased EC of groundwater in high-vulnerability areas (Figure 7a) shows groundwater pollution by industrial effluent, which is typically high in TDS [40,60]. Areas with high and extremely high pollution risks (Figure 7b) are frequently observed around agricultural and urban areas. Such a geographical distribution confirmed the result above. For the evaluation of global groundwater quality, the EC of groundwater should not exceed 2700 µS/cm [61]. Hazards in the research area provide signs of a very considerable risk of contamination, primarily from south to north.

Overall, the results of our current study revealed that the survey area is moderately vulnerable. These results are likewise confirmed by other authors in other regions in Morocco with a similar background, such as the results of an electrical tomography survey conducted by Salhi et al. that used the GALDIT approach and showed that the high-vulnerability area accounts for approximately 2.9% of the total area of the Ghiss Nekkor alluvial aquifer [58]. Additionally, vulnerable wells in the Ghiss Nekkor plain are located in the north and are characterized by high levels of salinity and Cl/Br and Cl/Na ratios close to seawater (Cl/Br = 650; Cl/Na = 1.2) [62]. Similarly, two classes of vulnerability are more notable in the coastline of Essaouira when calculated using the DRASTIC method. The results obtained using this method showed a high (22%) to very high (7%) vulnerability to groundwater pollution, which can be explained by the low slope of the cover, the existence of porous formations, and the shallow aquifer [63].

Research using the DRASTIC vulnerability model and nitrate contamination in Morocco indicates an increased risk of groundwater pollution from seawater intrusion and human activities; the most vulnerable areas are located alongside agricultural areas as well as villages and cities. This causes a serious environmental problem for the groundwater reserves in the coastal areas of Morocco [64,65,66,67,68]. Consequently, in the Ghiss Nekkor alluvial aquifer, our results show that the levels of EC generally exceed the WHO recommended limits. Additionally, nitrate levels were found to exceed recommended limits in the northwestern part of the aquifer. The output of the DRASTIC model has proven to be one of the most successful because of its performance and convenience of use. This shows that the pollution risk map is valuable information for making decisions related to the Ghiss Nekkor alluvial aquifer. To mitigate the future degradation of this groundwater and overcome the risks of pollution, we recommend controlled pumping, especially in the northern part, where the risk of marine intrusion is increasingly aggravated, to avoid the salinity of the water. Additionally, fertilizers and pesticides must be prohibited on agricultural land at particular times of the year.

## 5. Conclusions

In this research, the groundwater vulnerability of the alluvial aquifer Ghiss Nekkor was assessed using the DRASTIC model integrated into environmental GIS, considering seven hydrogeological properties. The DRASTIC vulnerability mapping categorized the region into five categories: very high (158–175), high (141–158), medium (124–141), low (107–124), and very low (90–107) vulnerability. The results showed that the areas under the agricultural fields in the northern part of the alluvial aquifer are vulnerable to increased pollution. The average vulnerability is around 137.7, calculated using the DRASTIC vulnerability index (DVI) formula. Furthermore, the low, medium, and high vulnerabilities represent 7.34%, 78.14%, and 14.5%, respectively, of the total area. The GIS technique provides an effective method to assess and analyze groundwater contamination susceptibility. The DRASTIC model shows that the concentrations of NO_3_ and EC follow a relatively similar progression to the graduated ranges of the vulnerability indices, especially in the eastern and western areas of the coastal fringe of Al Hoceima Bay. The most significant risk of contamination arises in urban areas with nitrate concentrations levels found to exceed recommended limits, particularly in the northwestern part of the aquifer. This study shows that the selected method can be an appropriate tool for the benefit of water and local authorities, and other decision-makers concerned about managing groundwater resources.

## Figures and Tables

**Table 1:** Hydrological groups covering the meteorological station in the zone of study [50].

Hydrological Group	Soils	Equation
B	Sandy—Sandstone—Sandy Loam.	R = (P − 15.05)^2^/(P + 22.57)
C	Sandy loam—Loamy sandy—Sandy clay—Nodular crusting.	R = (P − 19.53)^2^/(P + 29.29)

R: Annual net recharge in mm/year. P: Annual average rainfall in mm/year.

**Table 2:** Classification and weighting results of different parameters of hydrogeological factors according to the DRASTIC classification and weighting values.

Drastic Factor	Range	Rating	Weight
Depth to water table (m)	1.5–4.6	9	5
	4.6–9.1	7	
	9.1–15.2	5	
	15.2–22.8	3	
	22.8–30.4	2	
	>30.4	1	
Recharge (mm)	101.6–177.8	6	4
	177.8–254	8	
	>254	9	
Aquifer media	Massive Shale	2	3
	Thin Bedded Sandstone, Limestone	4	
	Massive Sandstone	6	
	Sand and Gravel	8	
Soil media	Peat	8	2
	Sandy Loam	6	
	Silty Loam	4	
	Clay Loam	3	
Topography (slope) (%)	0–2	10	1
	2–6	9	
	6–12	5	
	12–18	3	
	>18	1	
Impact of vadose zone material	Silt/Clay	3	5
	Silty Sand Clay	4	
	Sandstone	5	
	Sand and Gravel with Silt	6	
	Coarse Sand	8	
Conductivity (m/d)	4.1–12.2	2	3
	12.2–28.5	4	
	28.5–40.7	6	
	40.7–81.5	8	

## Data Availability

Not applicable.
